# Feasibility of Anti-reflux Gastric Bypass for Massive Paraesophageal Hernia in Obese Patients With Gastroesophageal Reflux Disease

**DOI:** 10.7759/cureus.45616

**Published:** 2023-09-20

**Authors:** Ahan Kayastha, Joseph Wasselle, Adam Wilensky, Joseph A Sujka, Rahul Mhaskar, Christopher G DuCoin

**Affiliations:** 1 Surgery, University of South Florida Morsani College of Medicine, Tampa, USA; 2 Internal Medicine, University of South Florida Morsani College of Medicine, Tampa, USA

**Keywords:** anti-reflux gastric bypass, heartburn, anti-reflux, gastroesophageal reflux disease, obesity, paraesophageal hernia, gastric bypass

## Abstract

Background

The objective of this study is to demonstrate the safety and feasibility of anti-reflux gastric bypass (ARGB) as a treatment for symptomatic massive paraesophageal hernias (PEH) in the obese population. Both gastroesophageal reflux disease (GERD) and PEH are particularly prevalent in the obese patient population, and obesity adversely affects the long-term outcomes of all anti-reflux procedures.

Methods

This is a single-center, retrospective review of 17 obese adults who underwent ARGB for the treatment of massive PEH between September 2019 and December 2021. Massive PEH was defined as >5 cm in a singular direction, and obesity as BMI ≥30 kg/m^2^. Patients without preoperative diagnostic testing were excluded. We reviewed and analyzed patient demographic data, postoperative symptom resolution, weight loss, and complications using descriptive statistics, change from baseline, and comparison of proportions.

Results

Sixteen of the 17 subjects were female. The median age was 48, and the median BMI was 39.10 kg/m^2^ (30.0-49.3 kg/m^2^). The average PEH size on imaging was 6.48 (H) x 6.25 (W) cm. The resolution of heartburn was 93.8% (p<0.001), and the resolution of nausea and vomiting was 80.0%. The mean postoperative length of follow-up was 9.12 months. Median excess body weight loss percentages at one, three, six, and 12 months were 16.43% (p<0.001), 35.92% (p<0.001), 40.64% (p=0.001), and 58.58% (p<0.01), respectively. Five patients experienced adverse events requiring additional intervention or hospitalization. There were no symptomatic hernia recurrences or mortality.

Conclusion

This study demonstrates that ARGB is feasible and potentially effective in treating symptomatic massive paraesophageal hernias in the obese patient population. Further investigation is needed to determine efficacy and long-term outcomes compared to standard surgical repair.

## Introduction

Over the last several decades, the prevalence and disease burden of obesity have risen steadily. Over a third of the U.S. adult population is currently classified as having obesity, which is operationally defined as a BMI of 30 kg/m2 or higher [1]. Obesity significantly increases the risk of developing both gastroesophageal reflux disease (GERD) and hiatal hernia [2-4]. Literature suggests that nearly 40% of patients with severe obesity have radiographic evidence of hiatal hernia, with obesity being proposed as one of the causal mechanisms for the development of reflux symptoms [2,5]. Given these associations, weight loss may play a role in resolving GERD. Today, Roux-en-Y gastric bypass (RYGB) and sleeve gastrectomy (SG) are two of the most commonly performed bariatric surgeries in the U.S. [6].

The current gold standard for the treatment of symptomatic paraesophageal hernia (PEH) is surgical repair via laparoscopy with the addition of an anti-reflux procedure, typically fundoplication [7]. However, long-term failure rates of anti-reflux procedures are higher in patients with severe obesity, leading to recurrent GERD symptoms [8]. Currently, there is no consensus on the ideal surgical management of symptomatic PEH in the obese patient population [9]. Considering the high recurrence rates associated with fundoplication in patients with an elevated BMI, we propose the use of the anti-reflux gastric bypass (ARGB) as an alternative for the surgical repair of symptomatic PEH with concomitant weight loss. The goal of this study is to demonstrate the outcomes of minimally invasive laparoscopic ARGB for the treatment of GERD in the setting of massive PEH in the obese population.

Anti-reflux gastric bypass should control GERD symptoms while decreasing the likelihood of hernia recurrence. It reduces intraabdominal pressure through weight reduction. This will decrease the pressure gradient between the chest and abdomen, thus theoretically reducing recurrence rates more than crural repair and fundoplication alone [10]. It is also believed that the roux limb will anchor the small gastric pouch into the abdomen, preventing recurrence [11]. Finally, the reduction of the size of the stomach with the creation of a gastric pouch reduces the amount of gastric acid exposed to the lower esophagus, thus treating the GERD symptoms [6].

Currently, ARGB is not recognized as an anti-reflux procedure by insurance providers in the treatment of GERD, regardless of obesity. We hypothesize that ARGB is a safe and feasible alternative to laparoscopic hiatal hernia repair with fundoplication for the reduction of GERD as well as symptomatic PEH recurrence in the setting of severe obesity. This article was previously presented as a poster presentation at the 2021 Scientific Session of the Society of American Gastrointestinal and Endoscopic Surgeons (SAGES) on August 31, 2021.

## Materials and methods

A single-center, retrospective review of patients with class II obesity and above undergoing massive PEH repair with ARGB from September 2019 to December 2021 was conducted in accordance with the research protocol approved by the University of South Florida Institutional Review Board (approval no. STUDY001736). The inclusion criteria consisted of a preoperative BMI ≥ 30 kg/m2, a singular hernia dimension ≥ 5 cm (defined as massive PEH) on preoperative diagnostic testing, and an age above 18 years. Exclusion criteria included patients without available preoperative diagnostic testing, immunosuppression, pulmonary hypertension, oxygen dependence, and class III to IV congestive heart failure.

Data collected included sample demographics (such as sex and age), preoperative weight and BMI, preoperative PEH size, pre and postoperative symptoms, operative time, and postoperative complications. Weight loss was measured at one, three, six, and 12 months of outpatient follow-up. Paraesophageal hernia size was measured for each patient via a coronal radiographic plane on either preoperative CT imaging or fluoroscopic contrast swallow imaging. The presence or absence of the following symptoms were noted from preoperative and postoperative clinic or hospital encounters: heartburn, regurgitation, dysphagia, cough, dyspnea, chest pain, and nausea or vomiting. Postoperative complications were defined as any deviation from the normal postoperative course requiring additional intervention or hospitalization.

Mean and standard deviation (SD) were calculated for continuous variables, and frequencies were calculated for categorical variables. The McNemar test was used to investigate the change in categorical variables (such as nausea or vomiting) preoperatively vs. postoperatively. The Wilcoxson signed-rank test was used to examine the difference in continuous variables (for example, weight) preoperatively vs. postoperatively. A p-value of <0.05 indicated statistical significance. Statistical analysis was done using SPSS Statistics version 26 (IBM Corp., Armonk, NY, USA).

All patients were initially enrolled for a preoperative evaluation and clearance at our bariatric center. They subsequently underwent a pathway program consisting of the same educational and preoperative behavioral interventions as patients without hiatal hernias undergoing RYGB. Operatively, ARGB is performed similarly to RYGB [11]. First, the hiatal hernia is repaired by bluntly dissecting the crura and the phrenoesophageal ligament, thereby freeing the esophagus. A posterior cruroplasty is performed to close the crural defect in a figure-of-eight pattern by using a 0-silk suture with teflon pledgets. Next, a small gastric pouch measuring 3 cm to 5 cm from the gastroesophageal junction and immediately distal to the left gastric artery is created and divided from the gastric remnant. A biliopancreatic limb, also known as the afferent limb, is then created by dividing the jejunum using an endoscopic linear stapler such that it measures roughly 75 cm. The jejunum distal to this division becomes the Roux limb, or the alimentary limb. Historically, the length of the Roux limb is determined as a function of required weight loss, with shorter Roux limbs utilized if weight loss is not indicated. At our center, Roux limb lengths of 75 cm, 100 cm, and 150 cm are utilized for patients with BMI <30, 30.0 to 34.9, and ≥35, respectively. Since all patients in this study had class II or III obesity, a Roux limb length of 150 cm was utilized. The relevant anastomoses are then created similar to RYGB and assessed for leaks with both methylene blue dye and air under saline irrigation.

## Results

Seventeen patients met the criteria for this study (Table 1). Sixteen of the patients were female, with a median age of 48 years. The median preoperative BMI was 39.10 kg/m2 (30.0-49.26 kg/m2). Mean PEH measurement was 6.48 (H) x 6.25 (W) cm. Median operative time was 286 minutes (149 to 468 minutes). The mean length of follow-up was 9.12 months (4-13 months). A biologic mesh (Gentrix 6-layer, ACell Inc., Columbia, MD, USA) was placed in two patients (11.8%). Five bariatric attending surgeons contributed towards this case series.

**Table 1:** Patient characteristics

Patient Characteristics	Values
Percentage of females	94.12% (16/17)
Median age (years)	48 (32-69)
Median preoperative weight (kg)	100.2 (72.6-134.3)
Median preoperative BMI (kg/m^2^)	39.1 (30.0-49.3)
Median preoperative excess body weight (kg)	45.2 (20.6-77.3)
Mean preoperative hernia size (cm)	6.48 x 6.25

Heartburn was reported by 94.1% of patients preoperatively, with 93.8% having symptom resolution postoperatively (p<0.001). Nausea and vomiting were reported by 29.41% of patients preoperatively, with 80% of patients having resolution postoperatively. One patient developed nausea and vomiting postoperatively that resolved within the first two weeks. Preoperative frequencies for the following symptoms were: regurgitation 52.9%, dysphagia 52.9%, chest pain 41.1%, dyspnea 17.6%, and cough 5.8%. Postoperative regurgitation and dysphagia were resolved in 88.9% of patients with preoperative symptoms; chest pain, dyspnea, and cough were completely resolved in all patients (Table 2).

**Table 2:** Symptom resolution ^1^ One patient developed nausea/vomiting postoperatively ^a^ Binomial distribution used

Symptom	Preoperative % (n)	Postoperative % (n)	Resolved % (n)	p-value
Heartburn	94.1 (16)	5.9 (1)	93.8 (15)	<0.001^a^
Nausea/vomiting	29.4 (5)	11.8^1^ (2)	80.0 (4)	
Regurgitation	52.9 (9)	5.9 (1)	88.9 (8)	
Dysphagia	52.9 (9)	5.9 (1)	88.9 (8)	
Cough	5.9 (1)	0.0 (0)	100.0 (1)	
Dyspnea	17.6 (3)	0.0 (0)	100.0 (3)	
Chest pain	41.2 (7)	0.0 (0)	100.0 (7)	

**Figure 1:**
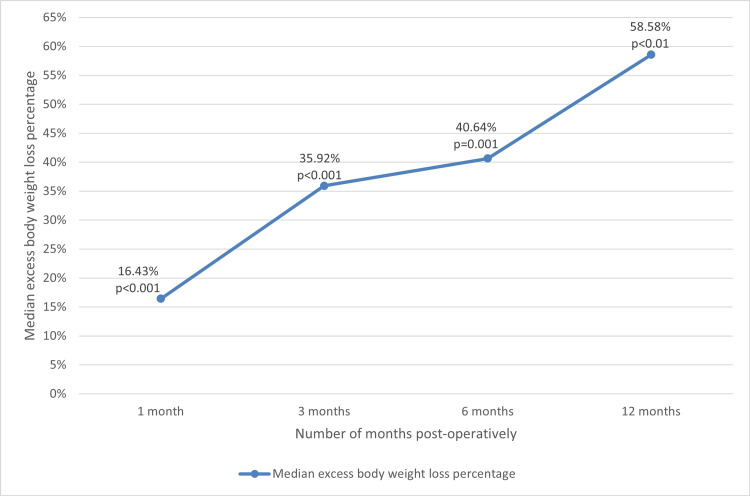
Median excess body weight loss percentage over time

Median weight loss postoperatively at one, three, six, and 12 months was 3.1 kg, 9.1 kg, 13.1 kg, and 20.5 kg, respectively. This correlates to a median excess body weight loss of 16.43% at one month (p<0.001), 35.92% at three months (p<0.001), 40.64% at six months (p=0.001), and 58.58% at 12 months postoperatively (p<0.01) (Figure 1). There were no symptomatic PEH recurrences or perioperative deaths noted in any patient.

Complications were noted in five patients (29.41%) within the follow-up period, which included two complications occurring during the hospital stay and three developing after discharge (Table 3). Using the Clavien-Dindo classification, the complications developing during the hospital course included one biliopancreatic limb obstruction (IIIb) and one anastomotic leak with mediastinal drainage (IIIb). The long-term complications developing after hospital discharge included one GJ limb obstruction (II), one gastrojejunostomy stenosis (IIIb), and one intermittent small bowel obstruction due to adhesions (IIIb). Of these patients, four required additional interventions (Table 3), and one individual had to be readmitted to the hospital. The median duration for long-term complications developing out-of-hospital was 30 days.

**Table 3:** Complications BP: Biliopancreatic, POD: Postoperative day

Complication	Number	Postoperative day	Location	Clavien-Dindo classification	Intervention	Outcome
BP limb obstruction due to intraluminal bleed secondary to idiopathic thrombocytopenic purpura	1	0	In-hospital	IIIb	Percutaneous endoscopic gastrostomy feeding tube placement	Discharged on POD #7 with improved thrombocytopenia and removed feeding tube
Anastomotic leak with mediastinal drainage	1	2	In-hospital	IIIb	Exploratory laparoscopy with mediastinal washout, mediastinal chest tube placement, wound vacuum device placement, and broad-spectrum IV antibiotics	Discharged on POD #21 after resolution of leak on repeat upper gastrointestinal series, removal of chest tubes and wound vacuum system, and completion of 14 days of IV antibiotics
Gastrojejunal limb obstruction	1	4	Out-of-hospital	II	Nasogastric tube decompression, bowel rest	Discharged with resolution of symptoms
Gastrojejunal stenosis	1	31	Out-of-hospital	IIIb	Esophagogastroduodenoscopy with dilation	Discharged with resolution of symptoms
Intermittent small bowel obstruction	1	15	Out-of-hospital	IIIb	Laparoscopic lysis of adhesions	Discharged with resolution of symptoms
Total	5 (29.41%)					

Patients were not routinely imaged for evaluation of hiatal hernia recurrence; however, four had imaging several months after surgery. These four patients were imaged at four, eight, and nine months. Three of the four had no radiologic evidence of recurrence, and one had a small recurrent asymptomatic hiatal hernia that measured 3 cm in length.

## Discussion

The management of GERD in the setting of massive PEH in the obese patient population is difficult due to the adverse effect that obesity has on patient outcomes, particularly recurrence [8]. We found that surgical management of this complicated disease process with ARGB was overall feasible and potentially effective. Gastroesophageal reflux disease was the most frequent symptom of those presenting with massive PEH, and this was significantly reduced by the ARGB. In addition, these patients experienced significant weight loss throughout the follow-up period. This has the theoretical advantage of reducing recurrence rates compared to standard PEH repair with fundoplication alone, in addition to the other health benefits of bariatric surgery, including reduced all-cause mortality [12]. Two patients in the series were revision patients with prior failed hiatal hernia repair with fundoplication due to recurrent hernia with refractory or worsening GERD symptoms.

The symptomatic hernia recurrence rate in our series of patients was zero; however, patients were only re-imaged if symptomatic. We found that of the 17 participants studied, a subset of four patients had imaging performed for other reasons, with one out of four having a radiological recurrence. The patient with the recurrence was reimaged at three months postoperatively as part of the workup for pyelonephritis, which demonstrated an incidental finding of hiatal hernia recurrence. The recurrence in question was a 3 cm hernia, and the patient did not have any reflux symptoms postoperatively. This finding is in comparison to a relatively high radiographic recurrence rate between 15% and 66% for standard PEH repair with fundoplication, although symptomatic recurrence is lower [13-16]. Other studies have examined recurrence in the setting of hiatal hernia repair and gastric bypass but not in the setting of massive hiatal hernias [17,18]. Our ability to detect recurrences is limited by the relatively short mean follow-up, and studies with longer follow-up and regular imaging will be needed to fully characterize the rate of recurrence in this population.

Our findings are consistent with those of a 2014 study by Chaudhry et al. of 14 obese patients undergoing RYGB for symptomatic PEH, which demonstrated comparable complication rates, weight loss, and symptom relief, suggesting the safety and success of such an operation [19]. Their study also included quality of life (QoL) metrics and determined a 78% good to excellent QoL on the GERD health-related QoL questionnaire. Another small series by Brammerloo et al. of 13 obese patients undergoing RYGB for large PEH also reinforces the safety of this procedure [20]. This study suggests exceptional safety (8% complication rate) and similar symptomatic relief. Both studies, as well as ours, had similar patient demographics in terms of sex, age, and BMI. Although the data from our study and a few others currently in the literature suggest that ARGB for the treatment of GERD in the setting of massive PEH is safe and effective, prospective comparative studies between fundoplication and ARGB are required before more definitive recommendations can be made. A recent comparative analysis by DuCoin et al. supports that patients with obesity and large hiatal hernias when treated with ARGB had similar GERD resolution, lower hiatal hernia recurrence, and improved weight loss when compared with fundoplication [11].

There was no mortality associated with our series of patients, although there were complications requiring intervention or hospitalization in 29.41% of patients. To further characterize the complications, we utilized the Clavien-Dindo classification system, a well-validated and widely utilized system in the field of surgery that focuses on the therapeutic consequences of a complication and aids in the objective comparison of outcomes among different centers and surgeons [21]. Anti-reflux gastric bypass is inherently more complex than a fundoplication, given multiple anastomoses and the surgically altered postoperative anatomy. In our study, the complication rate for ARGB was similar, but slightly higher, to that of standalone fundoplication, which is reported in the literature between 6% and 26% [13,22,23]. A recent study examining hiatal hernia repair and RYGB, but not massive hiatal hernias, found a complication rate of 26.3% within one year of surgery [17]. Perioperative outcomes for hiatal hernia repair with RYGB, such as overall morbidity, have been reported in the literature between 6.2% and 9.0% [24,25]. In a large study utilizing the United States Nationwide Inpatient Sample between 2004 and 2009, al-Hadad et al. found no evidence of increased risk of perioperative adverse events among patients undergoing concomitant hiatal hernia repair with RYGB when compared with RYGB alone, measuring several outcomes including deep venous thrombosis, pulmonary embolism, hemorrhage, injury to adjacent organs, postoperative fever or shock, etc. [26]. Moreover, the perioperative safety of RYGB has steadily improved over the last quarter century, with complication rates decreasing from 11.7% to 1.4% from 1998 to 2016 [27]. The authors admit that the complication rate reported in our study is higher than previously reported, which may be secondary to a small sample size. Additional studies with larger numbers of participants need to be conducted to assess the true complication rate of ARGB. In addition, patient selection and perioperative optimization in bariatric surgery are crucial components regarding outcomes. The authors expect that the true complication rate of ARGB will also decrease with perioperative optimization of comorbidities in high-risk patients.

Another more practical difficulty we acknowledge is that most insurers do not identify ARGB as a treatment for morbidly obese patients with GERD in the setting of large PEH. Further studies will help build a basis from which to have this lack of coverage reconsidered. Currently, these patients are only granted coverage for either an anti-reflux procedure that is prone to recurrence or to enter a bariatric program that can take months to complete while the risk of aspiration remains present.

Our study does have certain limitations. First, this study was retrospective in nature with a limited sample size, and a standardized survey to determine the presence of symptoms pre or postoperatively was not utilized. In the future, a standardized survey and QoL given both pre and postoperatively can be employed to categorize patients’ symptoms and their resolution more clearly. Additionally, a longer follow-up would have been preferable. Second, the study did not radiographically evaluate all patients for asymptomatic recurrence postoperatively. It is not the practice of our group to image patients without symptom recurrence; however, we recognize that it would be of distinct interest in this patient population as the radiographic recurrence of hiatal hernias can be as high as 24% [28]. In the future, radiographic evaluation of asymptomatic patients may be of benefit. Another note is that we used 5 cm as our definition of a massive PEH; however, there is no consensus on a definition of this, with some authors defining massive PEH as the entire stomach in the mediastinum [29,30]. The authors recommend that further studies with a larger sample size be performed that employ a QoL survey, utilize a recurrence algorithm, and potentially randomize patients between fundoplication and ARGB.

## Conclusions

Anti-reflux gastric bypass is a feasible and potentially effective alternative to treat symptomatic massive PEH in the obese population, with similar complication rates compared to fundoplication. While no conclusions about recurrence can be drawn due to the limitations discussed above, our study adds to the growing body of literature suggesting that ARGB has excellent GERD symptom resolution rates and may be a superior alternative to standard anti-reflux procedures in the obese population due to concomitant weight loss. There is a need for comparative studies to further evaluate the relative benefit of ARGB over other anti-reflux procedures.
